# Enabling Dynamic Partnerships through Joint Degrees between Low- and High-Income Countries for Capacity Development in Global Health Research: Experience from the Karolinska Institutet/Makerere University Partnership

**DOI:** 10.1371/journal.pmed.1001784

**Published:** 2015-02-03

**Authors:** Nelson Sewankambo, James K. Tumwine, Göran Tomson, Celestino Obua, Freddie Bwanga, Peter Waiswa, Elly Katabira, Hannah Akuffo, Kristina Persson, Stefan Peterson

**Affiliations:** 1 Principal, College of Health Sciences, Makerere University, Kampala, Uganda; 2 Department of Paediatrics and Child Health, College of Health Sciences, Makerere University, Kampala, Uganda; 3 Department of Learning, Informatics, Management and Ethics, Karolinska Institutet, Stockholm, Sweden; 4 Department of Public Health Sciences, Global Health, Karolinska Institutet, Stockholm, Sweden; 5 School of Biomedical Sciences, College of Health Sciences, Makerere University, Kampala, Uganda; 6 Department of Medical Microbiology, School of Biomedical Sciences, College of Health Sciences, Makerere University, Kampala, Uganda; 7 School of Public Health, College of Health Sciences, Makerere University, Kampala, Uganda; 8 Department of Medicine, School of Medicine, College of Health Sciences, Makerere University, Kampala, Uganda; 9 Unit for Research Cooperation, Swedish International Development cooperation Agency (Sida), Stockholm, Sweden; 10 Department of Microbiology, Tumor and Cell Biology, Karolinska Institutet, Stockholm, Sweden; 11 Department of Women’s and Children’s Health, International Maternal and Child Health, Uppsala University, Uppsala, Sweden

**Summary Points**

- Partnerships between universities in high- and low-income countries have the potential to increase research capacity in both settings.
- We describe a partnership between the Karolinska Institutet in Sweden and Makerere University in Uganda that includes a joint PhD degree program and sharing of scientific ideas and resources.
- Ten years of financial support from the Swedish International Development Cooperation Agency has enabled 44 graduated PhD students and more than 500 peer-reviewed articles, the majority with a Ugandan as first author.
- The collaborative research environment is addressing Ugandan health and health system priorities, in several cases resulting in policy and practice reforms.
- Even though all Ugandan PhD graduates have remained in the country and 13 have embarked on postdoc training, remaining institutional challenges include developing functioning research groups, grant writing, network building at Makerere, and continued funding on both sides of the partnership.

## Background

The Bamako Global Ministerial Forum on Research for Health emphasised the importance of developing national health research capacity in low- and middle-income countries (LMICs) as a key element in the strengthening of these countries’ health systems [1]. In Africa, there are a limited number of skilled health researchers given the burden of disease [2]. The World Health Report 2013 called for renewed efforts to strengthen health research capacity towards universal health coverage. Many initiatives have supported African countries in strengthening their national health research systems, such as the Initiative to Strengthen Health Research Capacity in Africa (ISHReCA), the African Network for Drugs and Diagnostics Innovation (ANDI), and Research for Health Africa (R4HA) [3]. Many initiatives, generally originating from high-income countries (HICs), are often criticized for failing to strengthen, incorporate, and involve low-income partners in priority settings and publications [4,5]. Given the challenges raised by transdisciplinary and international collaborative research, it is interesting to analyse existing institutional partnerships [6–8].

**Fig 1:**
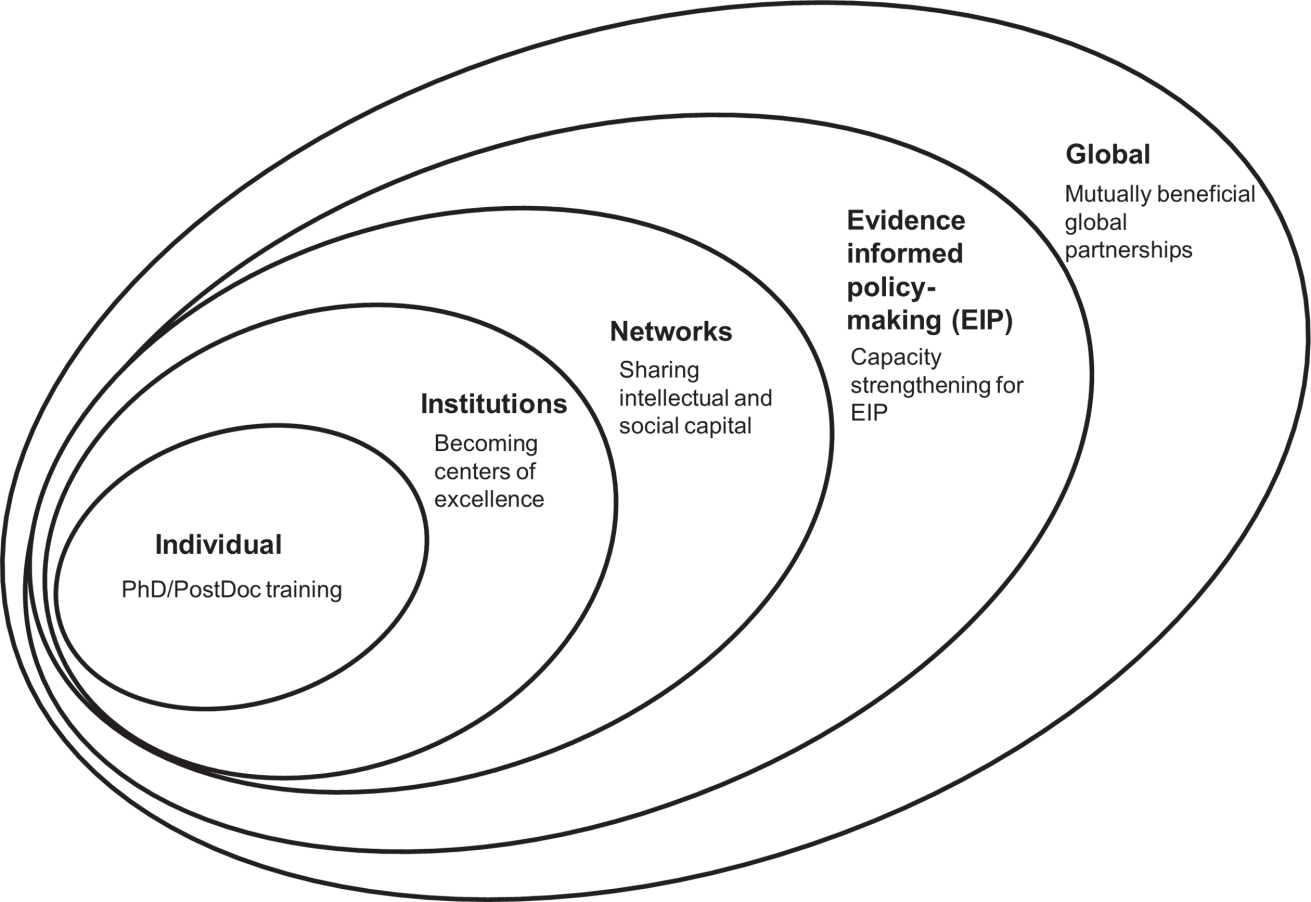
A framework for global institutional partnerships.

Several conceptual frameworks for capacity strengthening have been developed. The World Health Organization (WHO) offers a model focused on capacity development ranging from the individual to the supranational level [3]. The Alliance for Health Policy and Systems Research framework [9] focuses on capacity gaps for health systems and policy research. We combine elements of the two (Fig. 1) as a basis for presenting an innovative approach to health research capacity strengthening used in the institutional partnership between Makerere University (Mak) in Uganda and the Karolinska Institutet (KI) in Sweden, a partnership that also includes some scientists from Uppsala University. Our approach nests the individual student within the institutions and related research and decision maker networks, with the potential to contribute to evidence-informed policy making and mutually beneficial global partnerships. Spanning more than a decade, the partnership was developed on the foundation of an agreement financed by the Swedish International Development Cooperation Agency (Sida) [10].

## Establishing a Partnership between Makerere University and Karolinska Institutet

The research cooperation between Uganda and Sida was initiated in 2000. At the time, the Faculty of Medicine at Mak had a handful of doctoral candidates enrolled, primarily for the doctor of medicine by monograph dissertation. The supervision of students completing a by-coursework and publication PhD model was not done locally but by sending students abroad for the full length of study, with degrees awarded by the foreign university only. Sida’s support aimed to strengthen Mak University’s health research capacity through a partnership of senior researchers from Mak and Swedish institutions. Mak University identified priority areas of research and subsequently chose KI as its main Swedish partner for health research. KI, meanwhile, had experience in training foreign-based PhD students registered at KI alone, who spent some months per year at KI under so called “sandwich” arrangements. Sandwich arrangements refer to the practice of spending the major part of the research training time in the home country but traveling to the Swedish university for course work, data analysis, or supervised intensive working periods [11]. KI at the time had one joint degree with the University of Helsinki.

The first Memorandum of Understanding (MoU) establishing the institutional partnership was signed in 2003 (see S1 Text). Support from Sida to Mak has now covered three consecutive agreement periods from 2001 to date, and a fourth application is pending. Research areas identified by Mak, and mutually agreed upon, have been malaria (seven students), clinical pharmacology (seven students), tuberculosis (two students), mental health (nine students), cancers (three students), reproductive and child health (nine students), and health systems (seven students), with PhD trainees from the basic sciences, clinical medicine, and public health from both the university and the Ministry of Health.

## Developing a Joint PhD Degree

The major focus has been to train new PhD students in a joint PhD degree program, with the students working in a sandwich mode, spending most of their time in Uganda but traveling to Sweden for some of their specialized PhD courses, data analysis, thesis writing, and supervision [11]. This model was chosen to allow PhD candidates to continue their active home institution affiliation and to encourage research topics relevant to the national health systems. The degree is jointly awarded by both universities, based on double registration at both universities, dual supervision, and one defence with a joint examination committee. The student has to satisfy the requirements of both institutions in order to qualify for the joint degree award, conferred from either institution. The PhD is a 4-year, by-publication model, including one semester’s worth of PhD courses. Matters arising are handled by the respective university’s administrative staff and one research coordinator in each partner university. An issue of concern has been to ensure that degree certificates specify that this joint degree is awarded in collaboration with the other university so as not to give the impression that an individual has two PhDs. Subsequently, Makerere has also developed joint degree arrangements with Bergen University and Stellenbosch University, and Karolinska with Stellenbosch University and the National University of Singapore.

### Capacity Strengthening at the Individual Level

At an individual level, the Mak–KI partnership has been highly productive. The accomplishments are summarized in Table 1. These data highlight several important characteristics of the Mak–KI collaboration. First, we see that a majority of students chose the joint degree over a single-university degree. The collaboration has also been highly productive in terms of publications, with a majority of Ugandan first or last authors. In terms of subject area for research training, we see that Makerere prioritised this non-subject-tied funding to also include areas other than those with major funding (for example, HIV and malaria), such as degenerative diseases, cancer, mental health, and health systems research [12].

**Table 1:** Number of students and their outputs in the Mak–KI institutional collaboration.

Outputs			Mak–KI Institutional Partnership (2000–2013)			
			Joint degree (%)	Mak degree (%)	KI degree (%)	Overall (%)/range
Registered			42 (70)	6 (10)	12 (20)	60*
Sex	F		17 (40)	1 (16)	7 (58)	25 (41.7)
	M		25 (60)	5 (83)	5 (42)	35 (58.3)
Nationality	Ugandan		43 (87.8)	5 (10.2)	1 (2)	49 (81.7)
	Swedish				11 (100)	11 (18.3)
Completed programme by 2013			28 (66)^✚^	5 (83)^✚^	11 (91)^✚^	44 (73.3)
Median time in years (range) for completion of PhD			5 (3–9)	6.5 (5–8)	5 (3–6.75)	5 (3–9)
Number undertaking postdoctoral research			6 (21)	1 (20)	6 (55)	13 (30)
Publications in international journals			374	29	160	563
Publications with Mak student as first or last author						298 (52.9)

* Sida financed 48 out of the 60 students (joint and Mak degrees) with about 1.6 million Swedish kronor (SEK) per degree.

^✚^ From the original students registered to each degree. There are currently 15 students in the collaboration’s third phase (13 joint degree students, 1 Mak degree student, and 1 KI degree student).

The long-term arrangement has allowed time to develop partnerships at student, teacher, researcher, and administrative levels of the universities. This has created many spin-off collaborative activities in education and research. Long-term collaboration attracting additional resources in an environment in which research money is also available from large funders such as PEPFAR and the Medical Education Partnership Initiative (MEPI) [13–15] has reduced the risk of brain drain at Mak—to date, all the graduates have stayed in Uganda after completing their PhDs, with the exception of short or long postdoc periods abroad. We see these postdocs abroad as “brain circulation,” providing new skills and experiences as the postdocs have returned. Zero brain drain is a major achievement in a context in which the challenges in ensuring appropriate human resources for health research are considerable [2]. The sandwich arrangement also enabled male and female students alike to maintain social, cultural, and family lives while studying, which may be considerably harder in residential programs overseas.

Thirteen individuals have embarked on postdoc training after the PhD, either in sandwich mode based at Mak and at KI for different time periods, only at Mak, or elsewhere. As more people have graduated from the Mak–KI program and other capacity-building programs, there has been a rapid increase in demand for these positions, and the current Sida funding to Mak has a provision to support postdoc training. A particular challenge to Mak graduates in embarking on postdoc training is the strain on teaching and administration in the “home” department. Often, their absences for PhD training have necessitated colleagues taking over their tasks, and upon PhD completion the Mak graduates have to take up administrative and teaching duty. This may partly explain the lower proportion of Mak than KI graduates pursuing postdocs. To address this challenge, we are developing structured “sandwich postdocs,” whereby the PhD graduates remain attached to the home department, with periodic, mentored stays at the partner institution to enable them to transition into being independent researchers by acquiring, for example, technical, supervision, and grants management skills.

An additional 286 teachers and students have also been exchanged between the institutions, the majority on the Sida-funded Linneus-Palme program (teachers for a minimum of 3 weeks and students a minimum of 2 months), thus spreading the influence far beyond the research students and supervisors.

### Institutional Capacity Strengthening

Reflecting its cumulative efforts, Makerere was recently ranked fourth among African academic institutions and attempts to contribute to improved health outcomes in Uganda [16] while building academic networks to sustain progress [17]. An independent, external 2010 Sida evaluation of Mak’s collaborations with Swedish universities states that capacity development at Mak between 2000 and 2008 has been impressive [10]. Advances in research infrastructure have been considerable, including an Information and Communication Technologies Master Plan with Sida support, giving access to scientific journals, the establishment of a health and demographic surveillance site (HDSS), and the development of several research laboratories. Another important change for Mak was moving from the traditional doctorate of medicine by monograph to the publication-based PhD with public defence proceedings. Public defence of master as well as doctorate degrees is now the norm at Mak, and the majority of health science doctorates are by publication rather than by monograph. KI has also benefitted from the collaboration, which has strengthened research and education and led to several major spin-off grants as detailed below. Reciprocal learning opportunities include studies of antibiotic resistance and models for self-care in chronic illness.

Research management aspects are often inadequately described in the literature [6,18]. At Mak, the increased number of research students prompted financial and administrative reforms initiated from 2008–2009 with Sida support. The long-term funding and collaboration has enabled gradual adaptation and reform to fit the administrative, financial, and cultural realities of both institutions. Mak is building a strong, centralized research management system to keep track of all research funds brought in through various projects, which may have separate finance management structures. Importantly, the collaboration now also involves the respective grant offices at KI and Mak College of Health Sciences. This enabled environment has had spin-offs at network levels.

### Capacity Strengthening at the Network Level

At Mak and KI, collaborations within and between disciplines have been encouraged and have contributed to attracting new collaborators and funds. Examples of funders include the European Union, European and Developing Countries Clinical Trials Partnership (EDCTP)/Aeras, and the Gates Foundation. As a direct spinoff of a research project under the collaboration, Mak has also assumed leadership in the InDepth thematic group on maternal and newborn health. Furthermore, Makerere has many other South-South and South-South-North collaborations like the Wellcome Trust–funded African Institutions Initiative, the Makerere University/Uganda Virus Research Institute (UVRI) Infection and Immunity Programme, and the Malaria Capacity Development Consortium, which all contribute to the Makerere environment. In addition, there are strong collaborations through the MEPI collaboration with United States institutions and the Infectious Disease Institute’s broad international collaboration. Another strong partner is Johns Hopkins University [17]. In our experience, institutional strengthening through mutually beneficial partnerships may create effective and efficient research networks between HICs and LICs [19], which in turn offer the potential to attract substantial research funding.

### Capacity Strengthening at the Global Level

Research pursued within the Mak–KI collaboration has affected both national and international policies. Research results have affected neonatal and child health policy revisions in Uganda and internationally, for example, those concerning integrated community care management [20,21] and rapid diagnostics of multidrug-resistant tuberculosis [22]. Linkages between Mak and the Ugandan Ministry of Health (MoH) led to closer collaboration between Mak, KI, WHO, and the United Nations Children's Fund (UNICEF). The inclusion of MoH employees as PhD candidates in the program has facilitated this.

## Lessons Learnt

Effective, balanced international partnerships between universities in LICs and HICs are essential for tackling global health challenges by addressing priorities in ways adapted to the LIC context. We support the suggestion by Chu et al. on principles for such partnerships [5] and are happy to report actual results of 14 years of such collaboration. Mutual benefits include shared scientific resources, expertise and ideas, learning, and productivity. Deliverables include 44 graduated PhD students and a majority of publications with a first or last Ugandan author. Explanatory factors include a shared vision, trust, clear objectives, and continued funding over more than 10 years, as well as strong leadership on both sides, collaborative processes for prioritizing research topics, and selecting students to address Ugandan health and health system priorities.

However, challenges remain. At an individual level, there needs to be more investment in postdoc periods, including leadership, grant writing, and network building. At the institutional level, challenges include the need for further development of enabling environments such as functioning research groups, with seniors, postdocs, and students, and—because procurement and accountability procedures have taken a disproportionate amount of time during the collaboration—a supportive administrative and procurement system. Additional investment in core research infrastructure, such as population cohorts and a biobank, would also greatly enhance the capacity to do research. Sida supported information technology (IT) investment in fiberoptics and bandwidth expansion for the initial years, which dramatically improved email access. However, adding further bandwidth to allow stable web conferencing would help improve the crucial area of communication between students and supervisors and perhaps even more so the exchange between supervisors in the two universities, who co-direct a research area and are both main supervisors of the PhD candidate. Here, travel to meet in person is key initially, and communication can subsequently be upheld via telephone and internet. Web solutions are also increasingly being used for joint distance seminars. However, bandwidth issues have so far limited the quality of distance interaction. In addition, we have put in place a function of academic coordinator, one on each side. These people act as troubleshooters on small and large issues and have a major role in managing joint processes for applications and reporting to Sida. A joint steering committee has also been set up and had three meetings to discuss matters. Apart from managing challenges and identifying opportunities, we believe such functions and processes also contribute to maintaining respectful relationships, similar to what Redman-MacLaren reports from Oceania/Australia [23], so that cultural differences may be enriching rather than encumbrances [24].

## The Way Forward

Makerere has many strong collaborations and partnerships, which together shape the environment and contribute to the results reported. However, we believe the joint degree and the wide scope of the Mak–KI partnership demonstrates how strengthening health research capacity through institutional collaborations can transform teaching and learning for research in biomedical sciences, clinical medicine, and health systems strengthening. Our collaboration has built both partners’ individual, institutional, and network capabilities while being of national and international policy relevance. While the capacity and networks created have been able to attract large external project funding, Sida’s long-term commitment has been the key to bringing in aspects of capacity development, which are often lacking in project grants. We believe this collaboration offers approaches other universities and donors may want to use in development efforts. However, longer-term sustainability beyond the direct Sida support will depend on the viability of the educational and research collaborations established between the partner universities. The capacity developed may also be used to build capability in regional universities and bodies beyond Mak, where Swedish partners serve more to backstop Makerere. Ultimately, creating national funding lines for research in Africa’s growing economies will be the key to developing and sustaining sufficient national capacities. Sustained economic growth and considerable mineral wealth income in many African countries needs to also be invested in research and research capacity. The need for research capability in a populous country with rapid growth of the population, economy, and education sector like Uganda is very large indeed and will require sustained investment in research, as it does in high-income countries. This will likely have to include a large component of domestic funding.

## Supporting Information

S1 Text MoU for joint PhD degree. (DOCX)
