# Correction: Enabling Dynamic Partnerships through Joint Degrees between Low- and High-Income Countries for Capacity Development in Global Health Research: Experience from the Karolinska Institutet/Makerere University Partnership

**DOI:** 10.1371/journal.pmed.1001816

**Published:** 2015-03-31

**Authors:** The PLOS Medicine Staff

The ninth author's middle initials are missing. The correct name is: Kristina E. M. Persson.
