# Supplementary material for: Enabling Dynamic Partnerships through Joint Degrees between Low- and High-Income Countries for Capacity Development in Global Health Research: Experience from the Karolinska Institutet/Makerere University Partnership
Source: PLoS Med. 2015 Feb 3;12(2):e1001784. doi: 10.1371/journal.pmed.1001784 (PMC4315568; doi:10.1371/journal.pmed.1001784)
Supplement: S1 Text — (DOCX) [file pmed.1001784.s001.docx]

Supplementary web material- Memorandum of Understanding for Joint PhD degree.


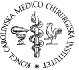

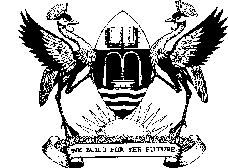


**Karolinska Makerere**

**Institutet University**

**MEMORANDUM OF UNDERSTANDING**

**Makerere University**

**Kampala, Uganda (hereinafter Makerere)**

**and**

**Karolinska Institutet**

**Stockholm, Sweden (hereinafter KI)**

**regarding**

**JOINT RESEARCH TRAINING TOWARDS**

**DEGREE OF DOCTOR OF PHILOSOPHY**

**ARTICLE I - INTRODUCTION**

Makerere University through its Faculty of Medicine and Karolinska Institutet have recognized the need to develop a joint PhD research training and a joint PhD degree to foster increased research collaboration. The present research collaboration between the universities is largely enabled through a grant from Sida/SAREC. The joint PhD degree is valid for students from both universities and is not limited to students funded from the Sida/SAREC grant.

The principle of the joint degree is to admit the students to a joint programme at the two universities and to fulfil the requirements of both universities, while making only one final examination, producing only one thesis and earning one degree.

The name of the degree shall be Doctor of Philosophy (PhD).

**ARTICLE II - PURPOSE**

The joint degree will strengthen the collaboration between senior researchers, facilitate staff development, improve administrative procedures, and foster internationalisation at both universities. A holder of the joint PhD degree will have the full recognition of the two universities and will become a future research fellow that can sustain and develop contacts between the two universities.

**ARTICLE III – ELIGIBILITY AND REGISTRATION**

(a) **Eligibility**

Candidates should fulfil the criteria for registration, training and examination at both universities and should undertake joint research projects with supervisors from both universities. See also Article VII, regarding Financial matters.

(b) **Application process**

The applicant will first register at her/his home university and then at the partner university. On the application forms for Makerere and KI respectively, the candidate should specify that he/she wishes to register for a joint research-training programme for a joint PhD degree according to this agreement. Prior to the registration, the application should be approved by the appointed coordinators at both universities.

(c) **Registration**

Registration will be handled by the normal administrative structures at Makerere and KI. In all regular documents both universities will note that the student is admitted for a

joint degree based on this agreement. Students starting at Makerere will first do a preliminary registration at Makerere for a maximum of one year. During this period supervisors from both Universities should be firmly identified, research-training courses followed, and the final research proposal developed. This final proposal will be vetted at Makerere as well as at KI and once approved the student will be fully registered. Students joining from KI should be required to follow preregistration preparatory training equivalent to that of Makerere.

**ARTICLE IV - SUPERVISION OF RESEARCH**

(a) **Requirements for supervisors**

There should be one supervisor at each university with equal responsibilities. In addition to these, further supervisors from either partner university, or any other university or research institute may be included. At KI, a PhD student must have at least one supervisor who is at the level of assistant professor (“docent”).

All supervisors should have a PhD degree or equivalent with at least two years additional research experience or be appointed to a position where one is qualified to supervise according to the university regulation.

(b) **Ethical Approval**

Both universities must give ethical approval of the planned studies, according to their respective regulations, before studies begin.

**ARTICLE V - PERIOD OF CANDIDATURE**

In addition to the year of preliminary registration required at Makerere, the programme should cover a total of three to five years full time or up to seven years part time studies. Prolongation is acceptable subject to application as per University regulations. If a PhD student wishes to register for the joint PhD degree programme after first having been registered only at the

home university, this must be done more than 2 years prior to examination

**ARTICLE VI – COURSE REQUIREMENTS**

The student must satisfy both universities’ course requirements which may be complementary or additional to those of either university

**ARTICLE VII - FINANCIAL MATTERS (a) Fees**

Candidates having Makerere as home university will pay fees to their home university, even while they are attached to the partner university.

Those having KI as home university will not pay tuition fees as this is not conducive with Swedish regulations. The costs incurred at Makerere will be covered by project grants as agreed by the supervisors

**(b) Research scholarship and travelling expenses**

Each successful student applicant must have secured financing from Sida/SAREC

grants or other funding sources prior to registration.

**ARTICLE VIII - CONTINUATION, WITHDRAWAL AND TERMINATION OF CANDIDATURE**

The prevailing conditions for continuation, withdrawal and termination of candidatures of the student's home university will apply. Students that leave the joint degree programme are free to pursue a degree at either university as existing regulations may provide.

**ARTICLE IX – EXAMINATION: THESIS/DISSERTATION**

Candidates from either university for the joint degree must have been admitted at both universities for a minimum period of two years before the degree is awarded.

The examination is carried out by an examining committee and an external examiner (Swedish “opponent”) of both the written thesis/dissertation and the oral defence (“*viva voce*”). Procedures are outlined below.

(a) **Written thesis/dissertation**

First the supervisors should certify - in writing as required by the bodies in charge of PhD examination at the two universities - that the thesis/dissertation is ready for examination. A joint examining committee will then be constituted by the regular appointing bodies of the two universities. The committee should consist of normally five, but if agreed three committee members, with at least one member from the partner university –either Makerere or KI –where the oral defence does not take place. One committee member should be external to KI and the medical faculty of Makerere. The three or five examining committee members (“viva-voce panel” at Makerere and “betygsnämnd” at KI) will make a preliminary evaluation of the thesis/dissertation. The thesis will be based on publications and manuscripts together with a written summary. If it is found acceptable, the committee will give the student permission to complete her/his thesis and undergo the oral defence (viva voce).

(b) **Oral defence of thesis**

For the joint PhD degree, it is proposed that each student undergoes one oral defence only, either at Makerere or at KI, with participation of the examination committee member(s) from the partner university (as specified above under IX (a)).

Apart from the three or five examination committee members, another external examiner (“opponent” in Swedish) will be jointly identified by the appointing bodies of the two universities. This external examiner/“opponent” must not be recruited from either Makerere or KI and will conduct the oral examination.

The oral defence will be a public (i.e. open to a general audience) 2-3 hour session that will be chaired by a chairperson from the arranging university who is not a member of the examination committee.

**(c) Examining committee meeting**

The student's performance during the oral defence, together with the written thesis, will form the basis of the examination committee's decision, which shall be made immediately after the oral defence on a vote in closed meeting with a simple majority of the three or five examination committee members. These three to five members will elect among themselves the person that will chair this closed meeting. The external examiner and the supervisor/supervisors may be present during the first part of the closed meeting to answer questions. The supervisors should leave the room before the voting.

**ARTICLE X - GRADUATION AND AWARD OF DEGREE (a) Graduation**

The student must satisfy:

- the graduation requirements for course work at both universities (qualifying examination);

- the written thesis/dissertation;

- the oral examination.

(b) **Certificates and Diploma**

The student should receive standard certificates (letter of award) from both universities In addition there should be one joint diploma issued by the university where the examination has been agreed to take place. This diploma should have the two university logos side by side, and statements attributed to both awarding bodies of the universities (Senate of Makerere University and the promoter at KI), title of the degree in the English and Swedish languages, explanatory note to accompany, student name, date of award, signatures of the two authorities, paper quality to be agreed upon and university seals to be embossed.

(c) **Transcript**

Each relevant official university transcript from either university should state that it is a joint degree between the universities.

**ARTICLE XI - USE OF INTELLECTUAL PROPERTY**

Ownership of any intellectual property (including, but not limited to, confidential information, know-how, patents, copyrights, design rights, rights relating to computer software, and any other industrial or intellectual property rights) developed on the base of a joint research programme during the course of this Memorandum of Understanding shall be vested in both parties to this Memorandum. Both parties shall have the joint right to determine the commercial exploitation and disposition of such intellectual property, and both parties shall make joint applications for the registration of the same. Before any registration or commercialization of any intellectual property takes place, the parties agree to reach a separate agreement covering issues such as exploitation rights and revenue sharing. Any publication regarding such intellectual property shall only be possible with the prior written consent of both parties, such consent should not be unreasonably withheld.

**ARTICLE XII - SPECIAL SITUATIONS**

Any dispute arising from the interpretation of this Memorandum shall be resolved jointly by the boards of postgraduate studies of the two universities, and in the case of non-resolution, by mediation between the Vice-Chancellor of Makerere and the President of KI.

**ARTICLE XIII - EFFECTIVE DATE AND TERMINATION**

This Memorandum of Understanding is valid for a period of five years and it is automatically extended by five years unless terminated by either university with a notice period of one year. If terminated, students enrolled under the joint degree programme will still graduate under the programme.

**Signed and delivered for and on behalf of Makerere University and**

**Karolinska Institutet**

**This Monday of 8 December 2003**

**President Vice Chancellor**

**Karolinska Institutet Makerere University**
